# Preventing Unintended Pregnancies and HIV Through Self-Care Interventions in East and Southern Africa: Findings From a Structured Review

**DOI:** 10.3389/phrs.2025.1607481

**Published:** 2025-03-04

**Authors:** Sofia Castro Lopes, Adriane Martin Hilber, Florence Secula, Yemurai Nyoni, Jyoti Shankar Tewari, Maria Bakaroudis, Renata Tallarico

**Affiliations:** ^1^ Independent Consultant, Cape Town, South Africa; ^2^ Swiss Centre for International Health, Swiss Tropical and Public Health Institute (Swiss TPH), Basel, Switzerland; ^3^ University of Basel, Basel, Switzerland; ^4^ Independent Consultant, Bulawayo, Zimbabwe; ^5^ UNFPA East and Southern Africa Regional Office, Johannesburg, South Africa; ^6^ UNFPA Rwanda Country Office, Kigali, Rwanda

**Keywords:** self-care, HIV, family planning, contraceptives, adolescents

## Abstract

**Objective:**

To identify promising interventions targeting young people in East and Southern Africa through self-care practices, with a focus on prevention of unintended pregnancies and HIV and develop four evidence-based self-care models.

**Methods:**

A structured literature review was conducted followed by a consultation with key stakeholders and youth networks from Malawi, South Africa, Zambia and Zimbabwe. Of the 2,890 published articles identified, 464 were fully reviewed and 59 were included in the final analysis along with 48 pieces of grey literature. A total of 31 youths participated in the consultation sessions.

**Results:**

Self-care interventions with high levels of feasibility, acceptability, and scalability included HIV self-testing, self-management of contraceptives, and self-awareness for improved and safer sex behaviours and sexual health. Key features of these interventions included the use of non-clinical environments, regular follow ups to reinforce practice, use of digital solutions, linkage to in person care, and participatory approaches involving young people from ideation to implementation.

**Conclusion:**

Self-care models that promote distribution, access, support through multiple mechanisms in non-clinical environments are more acceptable and more effective in reaching young people.

## Introduction

The Sexual and Reproductive Health and Rights (SRHR) of adolescents and youth are often compromised by stigma, misconceptions, sociocultural beliefs and norms, guilt and shame, resulting in limited or denied access to sexual and reproductive health (SRH) services [1]. Recent evidence suggests that self-care interventions and practices offer the potential to overcome several of these barriers by expanding and diversifying channels of access to commodities, information and services [2].

The potential of self-care interventions was further unveiled with the onset and aftermath of COVID-19 to ensure continuity of access to SRH services and information, and to ease the burden of in-person health service provision. From a health systems perspective, self-care interventions might contribute to strengthening the resilience of the health system and accelerating progress towards universal health coverage through increased coverage of primary healthcare services, as well as supporting the continuity of healthcare during emergencies or humanitarian crises [3].

According to the World Health Organization (WHO) self-care is: “the ability of individuals, families and communities to promote health, prevent disease, maintain health, and to cope with illness and disability with or without the support of a healthcare provider” [4]; and, self-care interventions are: evidence-based, quality tools that support self-care. These include medicines, medical devices, counselling, diagnostics and/or digital technologies that can be accessed fully or partially outside of formal healthcare facilities. Depending on the intervention, they can be used with or without the support of health workers. The scope of these definitions show that self-care can be applied to a variety of health conditions encompassing an array of healthcare interventions from health promotion, disease prevention and control, to rehabilitation and palliative care.

There are three domains of self-care, namely self-awareness, self-testing and self-management, with evidence-based actions under each domain for improving health and wellbeing. Another key feature of the WHO definition is the centrality of the individual(s) and their ability to take action (or not) to preserve, manage and respond to their own health needs, conditions or symptoms. It empowers individuals, families and communities by enhancing their agency, informed choices and decision-making. An underlying aspect of the emergence of self-care interventions, facilitated by advancement in health information and technology, is the shift in the healthcare paradigm, previously heavily reliant and under significant control of healthcare providers to a model where beneficiaries can self-determine healthcare interventions and support [5].

In the East Southern Africa (ESA) countries, youths face high risk of sexually transmitted infections (STIs), including Human Immunodeficiency Virus/Acquired Immunodeficiency Syndrome (HIV/AIDS), and early and unintended pregnancy resulting from the lack of access to information and contraceptive services as well as being denied access to appropriate healthcare, in particular sexual and reproductive healthcare, when required. Vulnerability is particularly high among young people who are out of school, live in rural areas, with disabilities, identify as LGBTQIA+, forced into early marriage, migrants, refugees and/or live in townships or slums. In recent years, the COVID-19 pandemic and the emerging conflicts in this region aggravated the vulnerability of youth to HIV infections and early and unintended pregnancies and other poor SRH outcomes in part by the incapacity of health systems to respond [3].

Although there is a recognition of the potential of self-care interventions [4, 6] and many promising self-care interventions focusing on specific SRH services are being implemented in Sub-Saharan Africa (SSA), there is limited knowledge about the feasibility, acceptability and scalability of approaches to implement comprehensive self-care interventions to improve SRHR.

United Nations Population Fund (UNFPA) East Southern Africa Regional Office (ESARO), in collaboration with WHO and AIDSFunds, with funding from the Swiss Agency for Development and Cooperation under the Safeguard Young People Programme were seeking to develop models to implement SRHR self-care interventions at scale for adolescents and young people (AYP) with a focus on prevention of early and unintended pregnancies and HIV testing and counselling services. This review was commissioned to assess the feasibility, acceptability and scalability of self-care interventions for SRHR including HIV prevention for AYP in the ESA region. This review focused on identifying promising practices undertaken in public and private sectors to develop four evidence-based self-care models to take self-care interventions to scale for preventing unintended pregnancies and STIs.

## Methods

### Study Design

This study combined a structured literature review, and consultations with key stakeholders and youth networks from four ESA countries, namely Malawi, South Africa, Zambia and Zimbabwe. In addition, a panel of experts was created (hereafter Reference group) to discuss the findings of each phase of the work and support the selection and design of the feasible, acceptable and scalable self-care models tailored to the needs of AYP.

The first phase of the study involved a structured literature review of published and grey literature on self-care interventions, focused on identifying good practices and lessons learnt applicable to SRHR. Four feasible, acceptable and scalable self-care models for AYP emerged from the findings of this phase, which informed the consultation with the stakeholders and beneficiaries of the selected countries.

### Structured Literature Review

#### Search Strategy

The search was conducted in steps. The first step was a scoping review of self-care interventions and models implemented globally aiming to identify and explore innovative approaches which could provide lessons learnt for our topics of interest as well as key search words. The second step, was refined search focusing on SRHR and HIV issues in SSA, including in the four target countries. The search was conducted on PubMed, Scopus, Embase and Web of Science for articles published between 2015 and 2022. The combination of the following keywords was used: “self-care” OR “self-Management” OR “self-medication” OR “self-treatment” OR “self-examination” OR “self-injection” OR “self-administration” OR “self-use” OR “self-testing” OR “self-sampling” OR “self-screening” OR “self-diagnosis” OR “self-collection” OR “self-monitoring” OR “self-awareness” OR “self-education” OR “self-regulation” OR “self-efficacy” OR “self-determination”) AND (reproductive health [MeSH Terms])) AND (HIV/AIDS [MeSH Terms]) AND (“Sub-Saharan Africa”) AND ((”2015” [Date–Publication]: “2022” [Date–Publication])) AND (project OR programme OR intervention).

A third search was conducted on internet search engines (Google, Google Scholar) and of websites to identify grey literature, including technical reports and other program documents on self-care interventions in the four target countries. A snowball sampling approach was used to identify contacts that could potentially provide further information or clarity about the implementation of interventions captured from the literature or documents found through the online searches. Personal communication through email or phone calls were used to reach out to the identified contacts and collect additional information.

The results of all of these steps were combined, duplicates removed, and narrowed purposefully to document evidence of self-care on SRHR and HIV which could inform the definition of self-care models adjusted to AYP in the SSA region.

Prior to starting the search process, the research team, in agreement with the Reference group, decided to exclude the term “self-help” from the search key words. This decision was based on the broad spectrum of interventions that are included in self-help beyond the scope of this work. In addition, Comprehensive Sexuality Education (CSE) and Life Skills education were also excluded from this review as it was deemed covered by other reviews during the review period. Although CSE/Life Skills education have been shown to influence key aspects of self-care such as self-awareness, self-efficacy, and self-determination [7], particularly related to making informed SRHR choices, the success of a variety of models for in and out of school CSE have been demonstrated and scaled up in many settings [8], therefore, CSE was not included in the search. Similarly, “self-efficacy” was excluded from the search key terms. “Self-efficacy” is a psychological variable extensively researched and measured in the published literature, but in the context of this self-care review, it was included only when self-efficacy was directly linked to another self-care activity (e.g., self-efficacy for self-testing and/or self-management).

#### Screening

**FIGURE 1:**
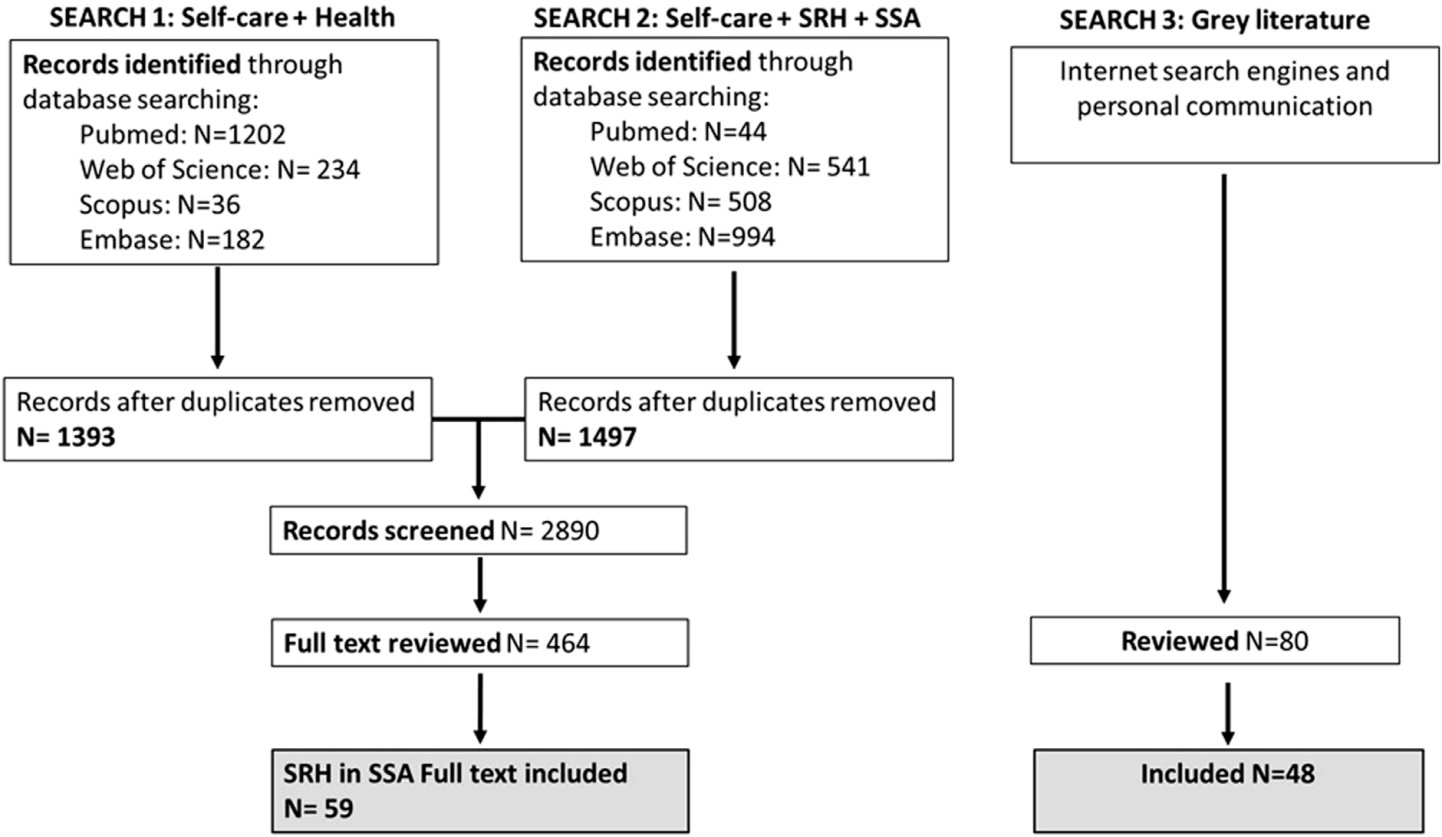
PRISMA diagram for the selection of reviewed articles and report (Worldwide, 2022).

A multistep screening process was conducted (Figure 1). Titles and abstracts of search results were screened by three reviewers: two reviewers focused on results for SRHR in SSA and one reviewer on the global search results - all scanned for promising practices and approaches that could be investigated and cross referenced further with existing practices from the ESA region. Discrepancies in screening results were discussed among the team until agreement was obtained. A similar process was conducted for the screening of full texts and inclusion of the final articles. The search and screening of the grey literature was conducted by one reviewer. Promising practices identified from the grey literature were included in the consultation and validation process.

### Eligibility and Inclusion Criteria

Quantitative and qualitative documents, written in English, between January 2015 and December 2022 were considered eligible. For the published articles the inclusion criteria were: 1) peer-reviewed; 2) full-text articles available; 3) clear self-care intervention described in detail; and 4) reporting on outcomes resulting directly from a self-care practice. Articles were excluded for the following reasons: 1) they were duplicates; 2) systematic, scoping, realist reviews and conceptual pieces such as commentaries; 3) when the self-care intervention was not clearly described; and 4) the studies did not report outcomes on the self-care practice.

It is worth noting that the reviewed articles, while informative, did not provide sufficient detail generally on the intervention strategies (i.e. success criteria) to inform the selection of potential feasible, acceptable and scalable models but were important to informing variations on models in respect to these aspects. Such information has been included in the discussion of the various models as appropriate.

### Consultation and Validation With Stakeholders and Youth Beneficiaries From Target Countries

A total of 31 youths from Malawi, South Africa, Zambia and Zimbabwe were convened in two separate digital consultations on the 17th and 24th of November 2022 to provide their inputs on developing SRHR-care interventions for AYP.

Prior to the consultations, the main findings and a description of the four feasible, acceptable and scalable SRHR self-care models were shared with the participants. During the consultations, the youths were asked to respond to six questions concerning self-care models and to discuss their responses with other young people. Key questions for discussion mainly covered feasibility and acceptability of self-care among young people and peers, preference for access points of self-care information and commodities, aspects making self-care user-friendly or otherwise, support strategies such as digital solutions, parent child communication, provider engagement, and peer support among others; as well as the interventions that young people recommend focusing on and other suggestions for self-care interventions (full interview script in Supplementary Material S1). The consultations were facilitated by the research team, the meetings were video recorded with the permission of the participants and written responses were provided to the research team by a focal point for each country after the meeting.

### Analysis

An extraction matrix matching the key components of the WHO conceptual framework of self-care [4] was used to systematically analyse the literature and capture the key features of self-care interventions. Using a Microsoft Excel document, the following information was extracted: Health area or disease; Description of self-care intervention (content); Target audience; Country or implementation setting; Self-care practices in terms of feasibility (ability to do the intervention); Self-care practice in terms of acceptability; Self-care practice in terms of scalability; Self-care practice in terms of sustainability; Brief description of impact and/or self-care outcome of interest and/or where information is available on effectiveness.

For the consultation, a comparative analysis of the answers provided to each question across countries was conducted. The key findings of the analysis were consolidated in a report and shared in the workshops. Participants reviewed the findings related to the existing models, pros and cons (from the literature) of the various approaches and discussed inherent assumptions about the acceptability and appropriateness of some of the models. The results of these discussions then informed the development and refinement of the proposed self-care models.

## Results

### Review Characteristics

The initial search yielded 2,890 published articles of which 464 were fully reviewed. Of the reviewed articles 76 specifically addressed self-care interventions on SRHR of which 59 provided evidence for SSA and were included in the final analysis and reporting of findings of this work. In addition, 80 pieces of grey literature were identified, of which 48 were fully reviewed and included in this study.

This article focuses on the findings of self-care interventions pertaining to SRHR implemented and/or tested in the SSA region, presented in Table 1. Table 2 summarises the findings from the grey literature about SRHR self-care interventions found in the target countries, namely Malawi, South Africa, Zambia, and Zimbabwe. The findings are described in the next section.

**TABLE 1:** Summary of included studies (African continent, 2022).

Health topics	Self-care practice	Aim, approaches & target population	Location & authors
SRHR			
Family planning			
Contraception	Self-administration	Comparing continuation rates of self-administering depot medroxyprogesterone acetate (DMPA-SC) vs provider/CHW administration and modes of distribution (clinical-based provider vs CHW) Women, 18 to 40 years	Malawi: Burke et al. [60]
Attitudes and behaviours	Self-efficacy	Promote positive ideation about family planning and increase contraceptive use through a digital health tool called Smart Client Women, 18 to 35 years	Nigeria: Babalola et al. [9]
	Self-awareness	Improve the knowledge and communication skills of adolescents and young people and their parents in relation to safe sex behaviours, sexual health through web-based programs and digital platforms and family planning Adolescents and caregivers; Highly vulnerable populations such as pregnant adolescents, young people with disabilities and LGBTQA + people	Tanzania: Millanzi et al. [10]
Medical abortion	Self-management Self-administration	Assess the effectiveness of self-managed medication abortion (misoprostol) with counselling prior to self-administration but without clinical support Girls (above 13 years) and/or adult women (18 to 49 years)	Nigeria and Argentina: Moseson et al. [84]; Nigeria: Stillman et al. [11]
Other SRH issues			
Mental health and resilience for vulnerable adolescents	Self-efficacy	Built trust, developed individual and group problem-solving skills, practice-based learning for healthy parenting and safer sexual practices, the importance of self-care and appropriate health seeking, and improving capacity to manage and save money through group sessions Vulnerable adolescent girls, 16 to 19 years (selling sex for a living, pregnant or with a child of 24 months or less)	Zimbabwe: Chingono et al. [12]
HIV and other STI			
HIV			
HIV testing	Self-testing	Increase HIV self-testing through different distribution and information sharing modalities, including door-to-door, secondary distribution in ANC visits, non-clinical sites, pharmacy, peers and through digital platforms Most studies targeted the under-tested population including the young population, generally above 15 years	South Africa: Adeagbo et al. [13]; Adeagbo et al. [14]; Deville [87]; Janssen [15]; Lebina [16]; Lippman [17]; Martinez Perez [18]; Pai [19]; Shapiro [20]; Sithole [21]; Sithole [22]; Tanser [23] Kenya: Marwa [24]; Mugo [25]; Pintye [26]; Gichangi [27]; Wilson [86]; Agot [88]; Drake [28] Malawi: Choko [29]; Choko [30]; Dovel [31]; Indravudh [32]; Indravudh [33]; Nichols [34] Uganda: Horvath [35]; Okoboi [36]; Matovu [37] Zambia: Chanda [38]; Mulubwa [99]; Phiri et al [39] Lesotho: Amstutz [40]; Amstutz [41] Nigeria: Iwelunmor [42]; Rosenberg [43] Tanzania: Hunter [44]; Hunter [45] Zimbabwe: Mukora-Mutseyekwa [46], Sibanda [47] Mozambique: Hector [48]
PrEP	Self-testing	Increase HIVST in people using PrEP through health services and peers or mobile clinics Young (16 to 25 years) and adult women (above 18 years)	Kenya: Ngure et al [49]; Wanga et al. [50] South Africa: Adeagbo et al [51]; Rousseau et al [52]; Birdthistle et al [53]
People living with HIV			
Mental health	Self-awareness	Improve symptoms of depression, anxiety and stress and develop resilience strategies through sessions with group leaders or lay health workers Young and adult people	Uganda: Vancampfort et al [54]; Tanzania: Dow et al. [104];
ART	Self-awareness & self-management/self-medication	Improve mental health and adherence to medication through mobile phones and apps to share information, motivation messages and counselling Young people and vulnerable populations (e.g., MSM)	Nigeria: John et al. [55] Ghana: Abubakari et al. [56]
HPV			
HPV	Self-screening	Assess acceptability and sensitivity of self-screening tests for HPV by giving women written and/or verbal explanations in comparison with standard care (test performed by health provider). Cost effectiveness of self-screening Women, 25 to 65 years	Kenya: Swason et al (2018); Cameroon: Crofts et al. [57]; Tanzania: Bakiewicz et al [58]; Uganda: Campos et al. [59]

**TABLE 2:** A summary of target countries’ grey literature on self-care appropriate for youth (Malawi, South Africa, Zambia, Zimbabwe, 2022).

	Malawi (MLW)	South Africa (SA)	Zambia (ZMB)	Zimbabwe (ZIM)	Focus organizations
HIVST	Community-based and peer-led delivery of HIV self-testing in rural areas	HIV self-test kits distribution through a diversity of models, including digital applications aiming to reach under-tested populations	HIV self-test kits distribution through a diversity of models, aiming to reach high risk and under-tested populations	The Government’s initial intervention included facility-based health-workers to demonstrate how to perform a self-test and provide self-tests to clients, now in scaling up phase through community and peer workers approach	MLW: STAR Initiative (PSI) SA: Wits, STAR Initiative members, Global fund, MSF ZMB: Gov. of Zambia, STAR Initiative members/Unitaid ZIM: Gov. Zimbabwe, Unitaid, PSI, CDC, Grassroots Soccer Zimbabwe, FHI360
Injectable contraceptives	DMPA-SC provided by clinic-based providers and health surveillance assistants to women aged 18–40 years	Not applicable	Women who opted for self-injectable contraceptives during family planning group counselling, would receive additional follow-up by service providers over several months	Not applicable	MLW: PSI, FHI360, MSI Malawi ZMB: Government of Zambia, OPTIONS, IPPF/PPAZ, PATH, JSI, PSI and UNFPA
Medical abortion	Not applicable	Not applicable	Prescription of medical abortion drugs through private sector healthcare providers, to be accessed at pharmacies by women of reproductive age	Not applicable	ZMB: Marie Stopes Zambia, PSI
Self-sampling HPV	Women to self-collect specimens for HPV testing through a community based approach in rural areas	Participants self-collected a vaginal sample in a private room following a verbal explanation on how to collect the sample by a community health worker in low resource settings	Not applicable	Not applicable	MLW: University of North Carolina (UNC) Project; SA: National Cancer Institute (SA), University of Cape Town, Columbia University and Cepheid Inc.

### Findings on SRHR Including HIV Self-Care Interventions in SSA and Target Countries

#### Self-Administration of Injectable Contraceptives

In the area of contraception, self-injection of contraceptive Depot medroxyprogesterone acetate - subcutaneous (DMPA-SC), emerged as a prominent self-care intervention. Self-administration of DMPA-SC after a brief explanation showed better outcomes including high acceptability and feasibility levels among users when compared to clinical provider or community health work administration. Successful implementation was also found among the youth, due to the confidentiality offered by self-administration, and its association with improved contraceptive continuation rate, compared to clinical based distribution, due to reduced access barriers. There was additional evidence on improving the contraceptive method-mix [60–75].

Operational advantages: It facilitates the scale up of the intervention, especially in hard-to-reach areas; higher perceived confidentiality by users; reduction of barriers leading to contraception discontinuation.

Countries covered by the literature: Malawi, Zambia and Uganda.

#### Self-Awareness for Family Planning and Other Bundles of SRHR Information and Services

Several interventions targeted the improvement of self-awareness for family planning or other bundles of SRHR information and services. These interventions were tested with different target audiences including women of reproductive age, young people, or vulnerable adolescent girls and young women. Promising self-care practices that emerged from these models were self-learning through innovative educational approaches mobilising either digital tools (such as mobile-phone based dramas, digital self-care applications, online platforms - especially during the COVID-19 pandemic) or innovative pedagogies (e.g. direct and hybrid problem-based pedagogy, problem-solving skills, practice-based learning). Most of these interventions aimed at attitudinal and/or behavioural change of the target audience towards safe sex practices and family planning in general. Several of these models were informed by key health psychology and behaviour change theories (behaviour modelling and soft skills development) and were measuring psychological variables such as self-efficacy or self-reported intention to engage in safe sex. Most interventions were considered feasible and acceptable by the participants and the authors concluded that they offer potential for scalability at community level. For those interventions most robustly measured, it was demonstrated that they were effective at increasing the self-awareness of the target groups on safe sex practices and generated short-to-mid-term positive attitudinal change towards safe sex/family planning [9, 10, 12, 76–83].

Operational advantages: Potential to reach young people through digital solutions; mid-term sustainability of behaviour changes without repeated training.

Countries covered by the literature: Nigeria, Tanzania, Malawi, Zimbabwe, South Africa.

#### Self-Management of Medical Abortion

Models aiming to enhance self-management of medical abortion tested in SSA focussed either on the provision of service or enhancing access to service. One model compared the effectiveness and safety of self-managed abortion compared to clinician-managed medication abortion, which demonstrated comparable effectiveness results. Two other interventions tested access to abortion medication through private pharmacies. Promising practices include the purchase of abortion medication with high levels of self-management and acceptability reported among women however improved quality of information provided by drug sellers and the need to strengthen demand and supply strategies among those pharmacists are required in tandem with the availability of such medication [11, 84, 85].

Operational advantage: Possibly enlarge accessibility to medical abortion services, where legal.

Countries covered by the literature: Nigeria, Zambia.

#### HIV Self-Testing

The HIV self-testing (HIVST) interventions aimed to increase test uptake and most studies focus on exploring best distribution modalities reaching the most vulnerable. For AYP, peer-distribution is the most promising practice not only with better acceptability and higher linkage to care post-testing than door-to-door distribution, but also by increasing the chances of support or supervision while performing it. In all four target countries, the findings showed that non-clinical sites of distribution of the tests have been prioritized, namely through community-based platforms, workplace or peers, with evidence pointing to improvements in reaching the most vulnerable and under-tested populations, including AYP. Of the type of tests offered, oral HIVST reported higher efficacy and acceptability among younger people. Some studies looked at the use of mobile apps or use of social media in combination with other approaches to increase HIVST. These usually combined the provision of information and guidance during and after HIVST through different platforms and the distribution of the test in a convenient way for the participant. The interventions showed high feasibility, acceptability and testing rates among participants. Interventions that involve youths from the design to the implementation phases also showed increased HIVST by youths [13–48], [86–103].

Operational advantages: Effective in reaching underserved or hard to reach populations; can be easily integrated with other interventions; use of technology increases acceptance and feasibility by tackling access and confidentiality barriers.

Countries covered by the literature: Kenya, Lesotho, Malawi, Mozambique, Nigeria, South Africa, Tanzania, Uganda, Zambia, and Zimbabwe.

#### HIV Self-Testing and PrEP at Health Facility

The practice of offering HIVST through self-care intervention followed by (if required) Pre-Exposure prophylaxis (PrEP) at the health facility or community levels was shown in different studies to be highly acceptable and feasible for the participants. Along with HIVST, the security of receiving the guidance for use of PrEP by a provider proved efficacious for reaching younger populations and increasing chances of use by promoting self-confidence to manage self-care interventions particularly for the first-time users [14, 49–53].

Operational advantages: Applicable at health facility and community levels; increased chances of reaching young populations; tackles self-confidence barriers by having a healthcare support person.

Countries covered by the literature: Kenya and South Africa.

#### Self-Management and Self-Awareness of People Living With HIV

Several studies tested models for the self-management of mental health, HIV testing and antiretroviral therapy (ART) uptake among people living with HIV. The interventions studied included the practice of group counselling and peer mentoring platforms as well as mobile-apps based on health monitoring and information access. These practices were effective in improving overall mental health and HIV-related behaviours including adherence to ART, uptake of HIVST and testing more generally [54–56, 104–109].

Operational advantages: Use of technology increases feasibility, reach and acceptance by tackling confidentiality and self-confidence barriers; increased chances of reaching young populations; applicable at health facility and community levels; increased chances to facilitate uptake of a wide-range of HIV-related services.

Countries covered by the literature: Ghana, Nigeria, Tanzania, Uganda and Zambia.

#### Self-Screening for Human Papillomavirus (HPV)

Human Papillomavirus (HPV) self-care interventions aimed at both assessing the acceptability and feasibility of HPV self-screening to increase access and intervention coverage and to raise awareness on cervical health. While the practice of self-screening (or self-collection of sample) was considered acceptable and an effective route for reaching underserved communities, some findings suggest that feasibility might be reduced in some settings as most women preferred to be supervised by a nurse when performing the intervention [57–59, 110–116].

Operational advantage: Effective in reaching underserved populations.

Countries covered by the literature: Cameroon, Kenya, Malawi, South Africa and Tanzania.

## Discussion

Self-care interventions can help expand access to comprehensive SRHR services to all, and specifically for AYP. This review identified four promising self-care models for SRHR, for the prevention of HIV infections and unintended pregnancies among youth that could be implemented at scale in SSA, and specifically the ESA region. The models are: 1) Self-management of ART in integration with contraceptives services, 2) Self-management of unintended pregnancies through pills, emergency contraception and self-injecting DMPA-SC accessed through different channels, 3) HIVST with condoms distribution, and 4) Self-management of medical abortion.

Findings across studies suggest that HIVST increases testing uptake particularly among vulnerable populations such as AYP. Access to HIVST kits and to comprehensive SRHR information through a variety of channels addresses privacy, confidentiality and physical and cultural barriers often experienced by this population was affirmed in different studies. These findings are particularly relevant in high-HIV settings. Furthermore, acceptability and feasibility, as well as cost-effectiveness are enhanced when HIVST as a self-care practice is considered in complement with other approaches or existing strategies [29].

With regard to the prevention and management of unintended pregnancies, self-injection of DMPA-SC shows promising results in lowering discontinuation rates as well as increasing access particularly among those in most vulnerable situations or settings, as also shown in evidence from projects implemented on ground with government support in Zambia and Malawi [68, 71].

Self-awareness is core to self-care for the prevention and management of unintended pregnancies with a variety of interventions showing promising results in different contexts. Self-awareness seems to work in addressing misconceptions and myths, as well as for enhancing and ensuring access to quality information to support and enable decision-making processes to self-test and self-manage. This aligns with WHO Guidelines for Self-care Interventions for Health and Wellbeing 2022 [4], where interventions should promote an enabling environment for people to safely and effectively adopt SRHR self-care practices, including through empowerment processes such as access to information for making informed decisions.

The evidence gathered in this review shows that self-care practices when adequately embedded in the context can be highly feasible and cost effective for expanding access, availability and use of SRHR services, with potential for scalability and unburden of the healthcare systems [5], however the impact of these interventions on the health providers’ workload and motivation remains unknown, thus requiring further assessment [117]. Furthermore, self-care interventions that are dependent on volunteerism can be difficult to implement and or sustain.

Despite the context specificity of self-care interventions, this review identified four key features that seem to affect the feasibility, acceptability and scalability of self-care interventions across different settings for AYP. There key features are:

1. SRHR Self-care interventions outside clinical spaces with ‘support persons’ could be hugely beneficial: Self-care interventions that are based in outside clinic spaces and that combine different access modalities to improve awareness, self-testing and self-management better address the needs of AYP in the SSA and ESA region. Also, it was noted in many studies that for self-care interventions that are outside clinic spaces the inclusion of a ‘support person’ in proximity or reachable can improve the confidence, engagement, and also establish the linkages to care when needed. Furthermore, increased regularity of interactions with the population group that the self-care interventions are trying to reach, including through phone calls, digital solutions and provider follow up seem to improve engagement and consistency in self-care practices.
2. Digital solutions are useful for attaining scale: Digital tools and interactive platforms address key issues of confidentiality and privacy for SRHR of AYP. However, its use is limited in low-middle income countries due to limited connectivity and unaffordability of data. Also, self-care digital solutions seem to benefit from having a ‘support person’ (as is the case for self-care interventions outside the clinic spaces) to improve the confidence of young people. Evidence also suggests that these interventions are more successful when implemented in tandem with other access options such as a clear link/referral mechanism to the health services.
3. Access to products for ‘self-screening’ and ‘self-management’ as well as linkages to in-person care are important: Peoples’ lack of access to health products for self-screening and self-management is a major barrier for self-care and in many settings. The link between self-care and health services can take multiple forms, namely an associated person from the health system acting as point of contact or referral person, digital platforms that link to the health system and allow for example the transmission of self-test results, self-administration records, algorithms that support decision-making that link to referral, results of a computer programme, among others.
4. Designing and delivering self-care interventions for young people requires a participatory approach: Many studies point to the need for provider’s values clarification, and for addressing other structural barriers that hinder young people’s access to SRHR information and products. Using participatory methodologies involving youths may support the scope of interventions that are more adjusted to the real needs and contexts of young people such as the human-centre designed interventions found in Iwelunmor at al, 2022 [42] and Rosenberg at al, 2021 [43] studies.

Based on the findings of this review and in consultation of the Reference Group, the four self-care models were developed to an incipient level and shared with youths during the consultation process. A summary of the models developed with inputs from the youth consultation is available in Supplementary Material S2.

### Strengths and Limitations

To our knowledge, this is the first structured review on self-care for SRHR focusing on preventing unintended pregnancies and sexual transmission of HIV in AYP in ESA. The participatory approach involving youths yielded important insights and nuances to the self-care models that otherwise would have been missed, validating identified models and approaches as well as tailored them more closely to youth’s needs. Moreover, the inclusion of grey literature expanded the scope to include unpublished documentation of what works from the field, informing the design of interventions that can integrate, be coupled, or potentiate existing initiatives.

Unlike a systematic review, the structured review did not include all articles on family planning and HIV self-care related to young people as some were not appropriate or feasible in the low resource settings. Also, while it did not assess the strength of the evidence reviewed, the consultation process was instrumental in minimising the effects of this limitation by providing key insights on AYP preferences, acceptability and feasibility of the proposed models and refine the interventions accordingly. This is not a systematic and/or comprehensive review, and it is likely that some important approaches for self-care interventions for youths were missed. Furthermore, the selection of articles might have been biased for the purpose of the review. In addition, young people were not the target population of most interventions reported, nonetheless, people aged 15 years or above were included, and conclusions on best approaches could be retrieved or extrapolated. This however denotes a gap in the availability of evidence for self-care interventions for young people.

### Conclusion

This study identified models based on promising approaches that can lead to the successful implementation of self-care interventions to prevent HIV and other STIs as well as unintended pregnancies among AYP in ESA. The review found that models that promote distribution, access, support through multiple mechanisms in non-clinical environments are more acceptable to young people and thus have increased chances of reaching and engaging young populations.

Although digital solutions are preferred for matters of confidentiality, privacy, stigma reduction and time by young people, having alternative options linked to a trained health professional provides a sense of safety as well as increased linkage to in-person care if something goes wrong. This is also an important consideration given the existing digital divide in an effort to facilitate access to services for all. It was also observed that youth-centred designing and validation of the models is key.

The suggested models and promising approaches identified in this review can contribute to improve access to SRHR information and services among AYP in settings where these services are often limited or denied while also indirectly supporting the strengthening and increased resilience of the health system through reduced in-person service delivery.
