# Supplementary material for: Preventing Unintended Pregnancies and HIV Through Self-Care Interventions in East and Southern Africa: Findings From a Structured Review
Source: Public Health Rev. 2025 Mar 4;46:1607481. doi: 10.3389/phrs.2025.1607481 (PMC11913615; doi:10.3389/phrs.2025.1607481)
Supplement: Supplementary file 1 [file DataSheet2.PDF]

Supplementary material

Summary of the selected models

A brief description of the models is provided below with a summary of the key points on feasibility, acceptability and scalability discussed during the youth consultation.

MODEL 1: SELF-MANAGEMENT OF ART IN INTEGRATION WITH CONTRACEPTIVE SERVICES

This model aims to promote adherence and retention to treatment by supporting the transition to self-management of ART of adolescents and young people living with HIV, while also increasing access to a range of contraceptives contributing to improved viral suppression rates, prevention of HIV transmission, quality of life and wellbeing. The key aspects of this self-care model are integration of care in existing ART platforms/services, accessibility, and availability of commodities.

The youths considered that the best approach for self-management of ART is integration with provision of contraceptives is through peer and/or group support, a source of safety and comfort for young people. This should be followed up with digital support to save young people's time, ensure wider access and support their privacy. Parent-child communication should be prioritised to support young people's adherence, through provision of transport, motivation, and nutrition. The least preferred approach is service provider support, as youths perceive them as not available and always busy, and at times compromising confidentiality.

MODEL 2: SELF-MANAGEMENT OF UNINTENDED PREGNANCIES THROUGH PILLS, EMERGENCY CONTRACEPTIVES AND SELF-INJECTING DMPA ACCESSED THROUGH DIFFERENT CHANNELS

This model aims to improve and expand access and continuity of use of modern contraceptives by supporting self-management practices including self-injection or emergency pill use, available through different channels of distribution. The key aspects of this self-care model are diversifying channels for accessing modern contraceptives by girls and young women, availability of commodities free of charge and combined approach and mentorship activities.

Young people reported preference towards a model that combines a digital platform (for example, an App) and face to face support, ideally through a health worker from whom they can access products and schedule private appointments without a waiting period and with a guarantee of anonymity. For in-person interactions, each contact with a young person should be maximised so they can be informed and get all the services they need in an integrated way. For example, HIV self-testing and HPV testing. The model needs to offer a menu of options that young people can choose from, that eventually lead to integrated and comprehensive care for more acute needs. Although to be effective and usable, availability of contraceptives at no costs must be ensured. Personalised support should be complemented by App reminders and information using a robot or avatars (preferred over human reminders from a health worker). For reminders and additional support, young people suggested accessing a website with more information or a hotline that is anonymous and used for referral purposes. It was noted that parent-child communication should not be done directly for prevention and management of unintended pregnancies due to concerns about confidentiality and stigma.

MODEL 3: HIV SELF-TESTING AND CONDOMS DISTRIBUTION

This model intends to increase access to and uptake of HIV testing through self-testing as well as to other HIV and STI preventive measures in a youth responsive and enabling environment. The integration of condom distribution in the model creates an opportunity to promote prevention and prevention messages, while also preventing onward transmission from those who tested positive. It also allows to extend the messages and information to other STIs and to other contraceptive methods that will expand choices. The key aspects of this self-care model are distribution and accessibility of the HIVST kits, availability of the kits, provision of information, support and counselling, and linkage to care. These are shaped by the implementation context, resources, and infrastructure available.

On this model, youths showed preference for approaches that are provided by peers and include support groups. In contexts where digital support exists, it should be optimised especially for young people in urban areas. It is necessary to ensure that young people also have access to support from health service providers in the event that something goes wrong. Door to door service provision and outreaches coupled with awareness raising campaigns through digital platforms can increase demand and uptake of the services. Parents can be engaged to raise their awareness on self-care and create a more open environment for young people to seek help from when they need to access self-care interventions.

Model 4: Self-management of medical abortion

This model aims to facilitate access to medication for medical abortion through different dispensation modalities however by trained providers including in health facilities, over the counter at pharmacies and chemists. Girls and young women who chose to have a medical abortion will be offered accompaniment through a hotline or face to face with a health provider, to ensure the procedure is complete, and that side effects or complications can be managed quickly and professionally through referral to a nearby health care facility.

Youths showed resistance to this model due to the perception of high risk associated. It was suggested that significant levels of support from health workers (in a respectable and non-judgemental way) for young people to engage, especially to manage side-effects and psychosocial needs. This would entail strengthening training and capacity building, including values of health workers on how to provide support to young people in a non-judgemental way.
