# Supplementary material for: Preventing Unintended Pregnancies and HIV Through Self-Care Interventions in East and Southern Africa: Findings From a Structured Review
Source: Public Health Rev. 2025 Mar 4;46:1607481. doi: 10.3389/phrs.2025.1607481 (PMC11913615; doi:10.3389/phrs.2025.1607481)
Supplement: Supplementary file 2 [file DataSheet1.PDF]

Self-Care Models Country Consultations Template

Date: ____

Country: ____

Number of young people: ______________________

Age range: ____

Gender: ____

Thank you for taking time to participate in this consultation

The purpose of this consultation is to conduct a regional assessment of self-care interventions for SRHR in the East and Southern Africa region– with a particular focus on prevention of unintended pregnancies and HIV testing and counselling among young people aged 15-24 years, and to the extent possible for adolescents aged 10-14 years old. The regional review includes a report on the current situation as well as documenting best practices and/or promising practices undertaken both by public and private sector entities. The findings will inform the development of up to five interventions to be piloted and/or taken to scale in Malawi, South Africa, Zambia and Zimbabwe.

As the next step of this study, we need to receive feedback on the self-care models from young people in each country. As such, we have invited you to take part in this virtual consultation for 2 hours and 30 minutes as part of an advisory group. You have been selected as representatives of different sectors in the SRH space with expertise within the SRH field.

Your name and any other identifying information will not be used in connection with any of the information you provide. If we wish to include a direct quote attributed to you, we will request this and give you a chance to review and approve the quote.

Do you give your consent to contribute to this consultation: _________________?

INFORMATION LINKS ON SELF-CARE MODELS	
HIV self-testing and condoms	https://www.youtube.com/watch?v=D_IHm3p8RW0
Self-management of family planning including pills	https://www.youtube.com/watch?v=fZdy492f4vA<br>https://www.youtube.com/watch?v=mzgv3xSY0X0
Self-management of abortion	https://www.youtube.com/watch?v=-fCbTGs4aJI
Self-management of ART with FP	https://www.youtube.com/watch?v=HuAZ_kwbHGg

Key Questions for Discussion on Self-Care Models:

1. Is the self-care intervention feasible in your context? Would you or your friends consider doing practicing self-care if you needed it?
2. Where would you like to access the test/ contraceptive/ medications for self-care/self-management?
3. What aspects of the model are easy to do/engage with?
4. What is more difficult?
5. Which approaches (parent child communication; peer or group support; provider support; digital support; access to other services, etc.) are the most interesting or necessary to make the self-care model work for young people?
6. Do you think there are other approaches or self-care actions that young people in your context could do or are doing that could be supported?
